# On AC-Field-Induced Nonlinear Electroosmosis next to the Sharp Corner-Field-Singularity of Leaky Dielectric Blocks and Its Application in on-Chip Micro-Mixing

**DOI:** 10.3390/mi9030102

**Published:** 2018-02-28

**Authors:** Yukun Ren, Weiyu Liu, Ye Tao, Meng Hui, Qisheng Wu

**Affiliations:** 1 State Key Laboratory of Robotics and System, Harbin Institute of Technology, West Da-zhi Street 92, Harbin 150001, China; tarahit@gmail.com; 2 School of Electronics and Control Engineering, Chang’an University, Middle-Section of Nan’er Huan Road, Xi’an 710064, China; ximeng@chd.edu.cn (M.H.); qshwu@chd.edu.cn (Q.W.)

**Keywords:** micromixer, induced-charge electroosmosis, leaky dielectric block, debye screening, electrochemical ion relaxation, sharp corner-field-singularity

## Abstract

Induced-charge electroosmosis has attracted lots of attention from the microfluidic community over the past decade. Most previous researches on this subject focused on induced-charge electroosmosis (ICEO) vortex streaming actuated on ideally polarizable surfaces immersed in electrolyte solutions. Starting from this point, we conduct herein a linear asymptotic analysis on nonlinear electroosmotic flow next to leaky dielectric blocks of arbitrary electrical conductivity and dielectric permittivity in harmonic AC electric fields, and theoretically demonstrate that observable ICEO fluid motion can be generated at high field frequencies in the vicinity of nearly insulating semiconductors, a very low electrical conductivity, of which can evidently increase the double-layer relaxation frequency (inversely proportional to the solid permittivity) to be much higher than the typical reciprocal RC time constant for induced double-layer charging on ideally polarizable surfaces. A computational model is developed to study the feasibility of this high-frequency vortex flow field of ICEO for sample mixing in microfluidics, in which the usage of AC voltage signal at high field frequencies may be beneficial to suppress electrochemical reactions to some extent. The influence of various parameters for developing an efficient mixer is investigated, and an integrated arrangement of semiconductor block array is suggested for achieving a reliable mixing performance at relatively high sample fluxes. Our physical demonstration with high-frequency ICEO next to leaky dielectric blocks using a simple channel structure offers valuable insights into the design of high-throughput micromixers for a variety of lab-on-a-chip applications.

## 1. Introduction

Electrically driven fluid streaming (electroosmosis) and particle transport (electrophoresis) are the eternal focus of electrokinetics, having undergone an exciting renaissance in micro/nanofluidics [1], electrochemistry [2] and functional biomedical devices [3,4] over the past ten years. Electroosmosis of conducting saline solutions in microchannels under an electric field externally applied across the channel length direction is generally dictated by the authoritative Helmholtz–Smoluchowski slip equation, which is obtained by dealing with the electric double layer (EDL) as a thin capacitor skin covered at a sharp material interface between the liquid and solid material of a native surface charge layer (Please refer to Ref. [5] for recent theoretical descriptions of triple charge layers near conducting media, including an ultrathin surface charge layer of free and bond charges, Debye screening cloud of counterionic charges, and an extended space charge region in desalinated regions):

(1) $$u_{\mathrm{EO}}=-\frac{\varepsilon \zeta_{\omega }}{\eta }E$$

In terms of the solution dielectric permittivity *ε*, liquid dynamic viscosity *η*, and the native zeta potential *ζ*_ω_. Where *ζ*_ω_ of insulating charged sidewalls is determinable from the fixed surface charge density at the phase interface by the mathematical relation σ_free_ = ε *ζ*_ω_/*λ*_D_ with *λ*_D_ denoting the Debye screening length. This equation, revealed in the late 19th century [6], resolves the long-term contradiction on electrophoretic translation of charged colloids that are suspended in electrolyte solutions, where the sample particles are supposed to be exempt from the action of electrostatic forces, in that surface charge disappears at the outer edge of EDL under the condition of complete Debye screening. The polarized liquid layer near the phase interface is in fact a sheet of electrical dipoles [7], however, and can be driven into electrophoretic motion by Coulomb force, giving rise to a distribution of velocity discontinuity at the sharp material interface between two distinct phases (Equation (1)). In this way, ‘force-free’ electrophoretic transport of freely suspended particles readily takes place in saline solutions of charge carriers, due to electroosmosis fluidic stress that are adjacent to the particle surface [7,8,9,10]:

(2) $$u_{\mathrm{EP}}=-u_{\mathrm{EO}}=\frac{\varepsilon \zeta_{P}}{\eta }E$$

In terms of the zeta potential *ζ*_P_ at liquid/colloid interface. The negative sign in Equation (2) results from momentum conservation of fluid-structure interaction. The conventional linear electrokinetics do share some shortcomings, for example, they time-average to zero in AC electric field, so that DC voltage difference has to be employed, which can cause severe Faradic reactions on electrode surfaces that are placed at channel ends [11,12,13].

In fact, any normal component of electric field lines emitted from a solid object surface at the early time can induce Debye screening in conductive saline solutions, not necessarily the fixed surface free charge density caused by chemical adsorption [14,15,16,17,18,19]. Starting from this point of view, Ramos and co-workers [20,21,22] made the pioneering researches into field-induced double-layer charging on metal electrodes excited by low-frequency AC voltage signals, where the electric field in the normal direction is offered by the induced free charge on conducting electrode surfaces, and elucidated that the tangential field component outside the diffuse double layer can effectively drive its own induced counterionic screening cloud on the conducting surface of blocking electrodes to engender AC electroosmosis at field frequencies on the order of the reciprocal RC relaxation time for electrochemical polarization. Because the ionic charge layer emerges as a result of the applied AC electric field, both the induced free charge and tangential field component are sinusoidal functions of the time variable under Debye-Huckel limit, resulting in time-averaged electroosmosis flow even in a harmonically oscillating field [23,24,25]. Later, Bazant and Squires [26,27] indicated that a background electric field can act on its own induced free charge within the Debye layer at a polarizable phase interface, which is genuinely a more accurate physical description of nonlinear electrokinetic phenomena, and they coined the term ‘Induced-Charge Electroosmosis (ICEO)’ to delineate electroosmotic streaming on polarizable or highly-charged solid surfaces immersed in saline solutions, which has incorporated the physical concepts of AC electroosmosis/traveling-wave electroosmosis (ACEO/TWEO) on driving electrodes in standing-wave [28,29]/traveling-wave [30,31,32,33,34,35] electric fields, ICEO on bipolar conducting [15,36,37,38,39,40,41,42]/semiconducting [43,44]/dielectric [16,45,46,47,48] materials, and electroosmosis of second kind due to surface-conduction-induced bulk concentration polarization [49,50,51,52]. This new category of electrohydrodynamic flow shows great advantages in suppressing electrode reactions and bubble productions, as well as actuating faster fluid flows than conventional linear electroosmosis, by virtue of its second-order voltage dependence in time-varying electric fields [14,53,54,55,56,57,58].

Zhao et al. [43] have recently analyzed nonlinear electroosmotic streaming adjacent to semiconducting solid surfaces in direct contact with electrolyte solution, where an insulating condition is designated at the out edge of Debye layer under the approximation of negligible electrochemical ion relaxation at frequencies much lower than the characteristic relaxation frequency of double-layer capacitive charging. In practical experiments, it is genuinely much better to use higher field frequencies to produce ICEO for evading electrochemical reactions as far as possible, so that the low-frequency presumption used by previous works may break down and cannot well establish the flow field of ICEO on semiconductor surfaces at varying field frequencies.

To address this issue, we herein presented a set of mathematical formulations to reconstruct nonlinear electroosmotic slip adjacent to the sharp corner-field-singularity of leaky dielectric blocks that are embedded on channel sidewalls. The triangular-shaped semiconductor protrusions disposed along the microchannel have a finite value in both dielectric permittivity *ε*_b_ and electrical conductivity σ_b_ in analogy to that of the working fluids. Counterionic screening in the liquid domain is taken into consideration to account for the presence of Coulomb force within the Debye layer that drives ICEO slip on the polarizable surface, while we exclude the phenomenon of blocking of charge carriers at the sharp phase interface from the side of leaky dielectric block in current analysis, as has been previously adopted in Ref. [43]. The total current injected from the bulk fluid into the diffuse double layer is explicitly included in our mathematical model [59,60,61,62,63,64], which becomes significant as the applied field frequency *f* approaches, but still no more than the reciprocal charge relaxation time of the fluid bulk *f*_f-bulk_ = σ_f_/2πε_f_. In this way, our mathematical description of ICEO on semiconductor surfaces can work in a relatively broad frequency range, approximately from more than *f =* 10 Hz (for eliminating conventional electrokinetics) to less than *f = f*_f-bulk_ (for avoiding the onset of bulk ionic screening).

Based on the improved mathematical descriptions, it is discovered that solid block with a small conductivity and moderate permittivity can even actuate faster ICEO streaming flow at high field frequencies than ideally polarizable metal electrodes, in that the inverse double-layer relaxation time is raised to higher frequency values by the larger impedance of the leaky dielectric block. Due to the occurrence of evident electrochemical ion relaxation for the high frequency range, the unravel of high-frequency electroosmotic streaming adjacent to the sharp field-corner-singularity of semiconducting obstructions is very useful for some practical applications, including drug delivery, biomedical diagnostics, cell transport, and so on, where electrochemical reactions are unwelcome.

In addition, a computational model is developed to study the feasibility of this high-frequency vortex flow field of ICEO for micromixing. The influence of various parameters for developing an efficient mixer is investigated, and an integrated arrangement of semiconductor block array is suggested for achieving mixing at relatively higher fluxes. Our physical demonstration with high-frequency ICEO using a simple channel structure offers valuable insights into the design of high-throughput micromixers for lab-on-a-chip applications.

## 2. Methods

### 2.1. Design of the Chip Structure for Studying ICEO Next to Leaky Dielectric Block

**Figure 1:**
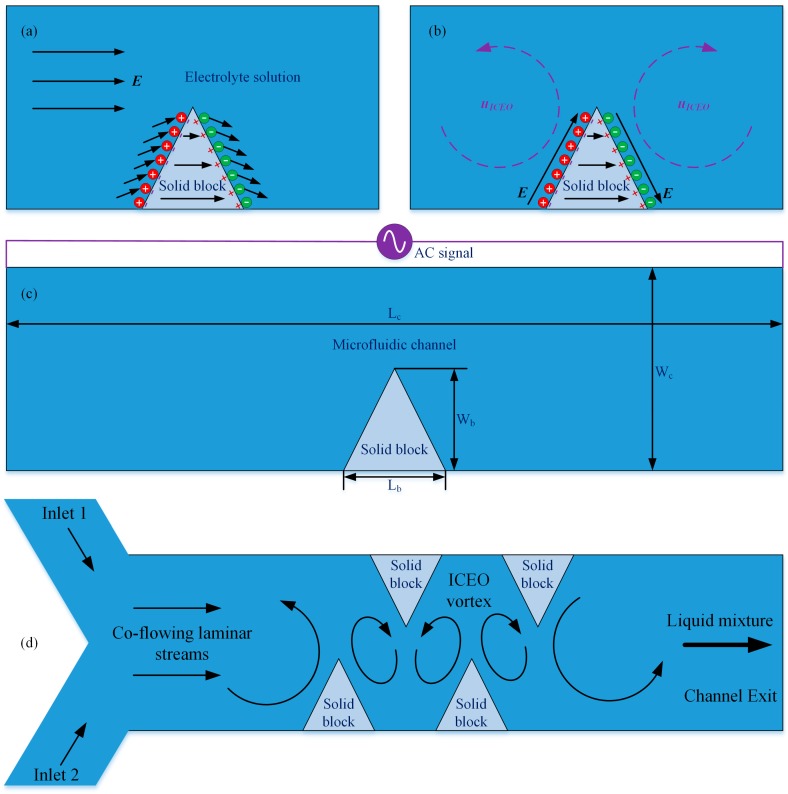
Schematic diagram of induced-charge electroosmotic (ICEO) next to triangular-shaped leaky dielectric block of a sharp tip with finite radius of curvature and its application to sample mixing in microfluidics. (**a**,**b**), Development of ICEO near a semiconductor obstruction of finite electrical conductivity and dielectric permittivity immersed in electrolyte solution, (**a**) at the early time, since the double-layer capacitance is in series connection with the leaky dielectric obstacle, Ohmic current from the bulk fluid charges both items at the same time, resulting in inclined electric field lines with respect to the solid surface that inject counterions into the Debye screening cloud; (**b**) after a characteristic double-layer relaxation time, Debye screening reaches the steady state, and no normal currents are allowed to flow into the thin boundary layer any longer, so that the electric field lines bend across the solid protrusion and forces the counterionic charges into induced-charge electroosmotic (ICEO) flow by exerting Coulomb force within the Debye layer; (**c**) Geometrical configuration of microfluidic channel embedded with leaky dielectric blocks for investigating ICEO near semiconductor protrusions; and, (**d**) An integrated design of electroosmosis micromixer taking advantage of the strong ICEO whirlpools induced adjacent to a series of sharp corners of leaky dielectric obstructions with staggered arrangement on opposing channel sidewalls.

In this work, we first conduct a simulation analysis on the flow behavior of ICEO near the sharp corner of semiconducting solid obstructions that are embedded on either side of channel sidewalls, as shown in Figure 1a–c. The fundamental chip structure for actuating ICEO adjacent to a leaky dielectric block in a straight microfluidic channel is presented in Figure 1c, where a semiconducting obstacle with a sharp tip at the top of solid structure is deposited at one side of channel sidewalls.

The radius of curvature of the geometric tip (*R* = 1 μm) is small enough to induce sufficient counterionic charges that are forced by the background high-frequency AC electric fields into induced-charge electroosmotic vortex around the semiconductor protrusion. The solid block of L_b_ = 50 μm in length and W_b_ = 20–180 μm in width, as well as the PDMS fluidic channel of L_c_ = 2 mm in length and W_c_ = 200 μm in width, are both assembled onto the surface of an insulating glass substrate. The block and channel share an identical height of H = 1000 μm to achieve seamless bonding for getting rid of water leakage. The main-channel is initially filled with electrolyte solution of electrical conductivity σ_f_ = 10^−5^–0.01 S/m and dielectric permittivity ε_f_ = 80ε_0_.

Once the optimal parametric space for actuating ideal ICEO vortex streaming next to an individual leaky dielectric block is determined by a series of steps of numerical studies in the presence of electrochemical ion relaxations, we validate the feasibility of this kind of high-frequency electroosmosis for realizing active electroconvection-enhanced mixing of nanoparticle samples in microfluidics with a new device design as shown in Figure 1d. Three branch channels, including two channel inlets and a single exit, are linked to three reservoirs, respectively (not shown). The channel entrance is of a ‘Y’ shape. Instead of single protrusion used in investigation of flow behaviors, one or multiple pairs of leaky dielectric blocks are arranged on opposing channel sidewalls with a staggered fashion in our electrokinetic micromixer.

In the simulation, the left inlet branch incessantly forces fresh aqueous electrolyte dispersing tracer nanoparticles into the fluidic channel, while the right inlet pumps pure buffer solution of same conductivity in the absence of dissolved substance. Due to the action of molecular diffusion effect, the phase interface between the two miscible laminar streams co-flowing along the channel length direction is expanded to a slight extent. At the same time, enhancement in mixing that is caused by passive stirring mechanism from the solid obstacles is negligibly small. As a result, a finite value of mixing performance on the order of 10% would be anticipated at the outlet port.

As soon as activating the function generator, a series of convective vortex pairs are produced in the transversal direction from field-induced polarization of the electrolyte/semiconductor interface, which intersects orthogonally with the side-by-side co-flowing laminar streams to generate zigzag trajectories of incoming tracer particles towards downstream by convective mass transfer, resulting in more efficient rotation and extension of the diffusing phase interface than the situation where merely diffusive mass transfer occurs. For these reasons, a delicate alternation of a few of ICEO whirlpools in opposite rotating directions can substantially accelerate sample mixing in microfluidic channels (Figure 1d).

### 2.2. Mathematical Model

We then mathematically formulate the physical descriptions of microfluidic mixing due to the action of electroconvective vortices, which are induced around the leaky dielectric blocks by an AC voltage difference externally are applied throughout the channel. Our analysis of ICEO near sharp corners of polarizable solid obstructions immersed in aqueous electrolyte is based upon the typical linear RC circuit theory of double-layer polarization at a sharp phase interface, under thin-layer approximation and small-voltage limit. We can tackle the mathematical problem by dividing the computational domain of the fluidic device into three correlating subsections, including the bulk flow, the Debye screening cloud and the leaky dielectric blocks embedded on channel sidewalls.

It is presumed that the applied AC voltage has a single Fourier component oscillating at the base frequency, i.e., ϕ(t)=Re(ϕ˜ejωt), where $\tilde{\phi }$ capped with a tilde symbol signifies the complex phasor amplitude of electrostatic potential ϕ(t) [65]. The tilde symbol ~ is dropped henceforward for convenience of expression. Within the liquid solution and leaky dielectric blocks, charge conservation in sinusoidal state is given by Equations (3) and (4), respectively [66,67,68]:

(3) $$\nabla \;((\sigma_{f}+j\omega \varepsilon_{f})\nabla \phi_{f})=0$$

(4) $$\nabla \;((\sigma_{s}+j\omega \varepsilon_{s})\nabla \phi_{s})=0$$

where the subscripts *i* = f or s represent the bulk fluid and solid domain, respectively. In this way, $\phi_{f}$ and $\phi_{b}$ denote the electrostatic potential phasor within the solution and block, respectively. Besides, σ*_i_* and ε*_i_* denote the conductivity and permittivity of the *i*th medium, respectively.

Under Debye-Huckel limit, we assume the concentration of charge carriers is homogenously distributed in the bulk region, so that Equations (3) and (4) are reduced to the Laplace equation of zero free charge density [69,70,71]:

(5) $$\nabla^{2}\;\phi_{f}=0$$

(6) $$\nabla^{2}\;\phi_{s}=0$$

At the semiconductor/liquid interface, it is necessary to provide conjugating conditions to guarantee the continuity of electrical current flux flowing across the Debye layer from physical constraints:

(7) $$\left(\sigma_{f}+j\omega \varepsilon_{f})n\cdot \nabla \phi_{f}=j\omega \frac{C_{D}}{1+\delta }(\phi_{f}-\phi_{b}\right)$$

(8) $$\left(\sigma_{b}+j\omega \varepsilon_{b})n\cdot \nabla \phi_{b}=j\omega \frac{C_{D}}{1+\delta }(\phi_{f}-\phi_{b}\right)$$

where ***n*** is the unit normal vector at the surface of the leaky dielectric block. Equations (7) and (8) implicitly indicates that the diffusion current and Ohmic current delicately counterbalance one another within the diffuse double layer due to the impenetrability of ionic species across the sharp material phase, so that the net conduction current vanishes in the thin layer adjacent to polarized surface of solid obstructions. In this way, the total current from either the fluid bulk (left hand side of Equation (7)) or the dielectric leaky block (left hand side of Equation (8) must be equal in value to the displacement current running through the double-layer capacitor skin (right hand side of Equations (7) and (8)), in order to meet conservation of current flux in AC electric fields.

The phasor amplitude of AC voltage signals is set to given values on both channel ends to represent an AC voltage difference externally imposed along the channel length direction:

(9) $$\phi_{f}=\;A\;(\mathrm{Channel}\;\mathrm{left}\;\mathrm{end})\;\phi_{f}=\;0\;(\mathrm{Channel}\;\mathrm{right}\;\mathrm{end})$$

The normal current flux disappears outside the native Debye layer induced by the fixed charge chemically adsorbed on insulating channel sidewalls, due to complete capacitive charging on non-polarizable surfaces:

(10) $$n\cdot \nabla \phi_{f}=0$$

Action of the tangential electric field component $E_{t}=E-E\cdot n\cdot n$ on the counterionic charges within the native and induced Debye layers results in linear and nonlinear electroosmotic slip velocity at the solid surface, respectively [72]:

(11) uslipEO=−εζfixedηRe(Etejωt)

(12) uslipICEO=−εηRe((ϕb−ϕf)1+δejωt)Re(Etejωt)

With the time-average counterparts given by:

(13) $$\langle u_{\mathrm{slip}}^{\mathrm{EO}}\rangle =0$$

(14) 〈uslipICEO〉=−εη12Re((ϕb−ϕf)1+δEt∗)

Since conventional linear electroosmosis time-averages to zero in a harmonic field (Equation (13)), we only take into consideration the effect of induced-charge electroosmosis in current analysis by inserting Equation (14) into the full Stokes equation as a slip-wall boundary condition on the surface of leaky dielectric blocks [73]:

(15) $$-\nabla p\;+\;\eta \nabla^{2}u=0$$

(16) ∇u=0

where *p* is the hydraulic pressure, and ***u*** the flow velocity vector.

Convection-diffusion equation is employed to describe the concentration distribution of sample nanoparticles inside the microfluidic channel under a combined action of Brownian motion, horizontal Poiseuille flow, and electroosmotic vortex next to the leaky dielectric block [74]:

(17) ∇(uc−D∇c)=0

where *c* denotes the local concentration of nanoparticles dispersed in the liquid suspension, and *D* the thermal diffusivity of chemical analyte, with the specific value of *D* = 10^−11^ m^2^/s determined from Einstein relation under the atmospheric condition for colloids of radius *r* = 20 nm.

### 2.3. Numerical Simulation

We make use of a finite element method (FEM)-based software package, COMSOL Multiphysics 5.2 (COMSOL Inc., Stockholm, Sweden), to analyze the induced-charge electroosmotic flow next to semiconductor blocks and its utilization in microfluidic mixing. The calculation procedure of ICEO and electroconvection-enhanced mass transfer in the fluidic channel is as follows. At first, the Laplace equation Equations (5) and (6) is calculated to obtain the electrostatic potential within the fluid domain and solid block, which are correlated. Electrostatic potential phasor ϕ=A and ϕ = 0 is prescribed on the left and right end of the microchannel, respectively. Conjugate conditions of Equations (7) and (8) are provided at the liquid/semiconductor interface to describe the capacitive charging of the electrical double layer at the phase interface by electric currents from both the solution and leaky dielectric block. Besides, normal component of total current vanishes $n\cdot \nabla \tilde{\phi }=0$ at other the insulating surface to close the electrical boundary-value problem.

Then, the full Stokes equation Equations (15) and (16) are calculated to obtain the flow field, with the mathematical expression of time-averaged nonlinear electroosmotic slip velocity (Equation (14)) being imposed on the block surface. Non-slip and no penetration n⋅u=t⋅u=0 are imposed on all of the channel sidewalls. A pressure-driven flow of a parabolic profile and surface-averaged flow velocity u_0_ is set at the two channel inlets for forward transport of the incoming particle samples, and zero pressure is applied at the channel exit.

Finally, transport Equation (17) is calculated for getting the mass distribution inside the fluid region under the synergistic action of diffusive and convective mass transfer, and any normal flux is forbidden on any sharp material interface (channel sidewall and block surface). Fluorescein of 40 nm in diameter with a diffusivity *D* = 10^−11^ m^2^·s^−1^ is employed in the simulation analysis, the concentration of which has a uniform value of *c* = 1 mol·m^−3^ at the left inlet and 0 mol·m^−3^ at the right inlet, respectively, while the normal diffusion flux vanishes at the channel exit, in order to reconstruct the actual situation for sample mixing in microfluidics.

We apply stationary solvers for the set of governing equations that are subjected to boundary conditions from physical constraints. The complex voltage phasor, fluid motion, and sample delivery are sequentially solved in a segregated manner.

The sharp tip of leaky dielectric block with a triangular shape has a radius of curvature about *R* = 1 μm, which is a reasonable value that is encountered in practical experiments. For benchmark modeling, it is discovered that the specific value of *R* exerts a significant effect on the ICEO flow field around the obstruction, and the magnitude of electroosmotic flow velocity increases with decreasing *R*. The reason behind the phenomenon of enhancement in fluid motion through making the block tip ‘sharper’ is caused by the domination of double-layer capacitive charging over polarization of semiconductor obstacles at the section of corner field singularity. In this sense, the value of *R* has to be controlled with great care in our numerical calculation. A minimum mesh size *R*/20 = 25 nm is set right at the block tip, with a maximum growth rate of 1.03 for grids extending to the bulk fluid, so as to confirm the grid-independence of simulation results.

### 2.4. Impedance Analysis

The electrical impedances of fluid bulk adjacent to the polarizable surface Z_f_, Debye screening cloud Z_D_, electrical double layer Z_EDL_ and the leaky dielectric solid block Z_b_ per unit area are as follows [75]:

(18) $$Z_{\mathrm{EDL}}=\frac{\lambda_{D}(1+\delta )}{j\omega \varepsilon_{f}},\;Z_{f}=\frac{W_{g}}{\sigma_{f}+j\omega \varepsilon_{f}},\;Z_{b}=\frac{R}{\sigma_{b}+j\omega \varepsilon_{b}},\;Z_{D}=\frac{\lambda_{D}}{j\omega \varepsilon_{f}}$$

Accordingly, the total electrical impedance of the solid/liquid phase interface is the sum of the former three components:

(19) $$\begin{array}{l} Z=Z_{f}+Z_{\mathrm{EDL}}+Z_{b}\\ =\frac{W_{g}}{\sigma_{f}+j\omega \varepsilon_{f}}+\frac{\lambda_{D}(1+\delta )}{j\omega \varepsilon_{f}}+\frac{R}{\sigma_{b}+j\omega \varepsilon_{b}}\\ =\frac{\frac{W_{g}}{\sigma_{f}}}{1+j\omega \tau_{f}}+\frac{\lambda_{D}(1+\delta )}{j\omega \varepsilon_{f}}+\frac{\frac{R}{\sigma_{b}}}{1+j\omega \tau_{b}} \end{array}$$

where $\tau_{f}=\frac{\varepsilon_{f}}{\sigma_{f}}$ and $\tau_{b}=\frac{\varepsilon_{b}}{\sigma_{b}}$ denote the charge relaxation time of fluid bulk and semiconductor block. A fraction of the applied voltage difference drops across the Debye screening layer of mobile counterionic charges, with the value of this factor given by:

(20) $$\alpha =\frac{Z_{D}}{Z}=\frac{\frac{\lambda_{D}}{j\omega \varepsilon_{f}}}{\frac{W_{g}}{\sigma_{f}+j\omega \varepsilon_{f}}+\frac{\lambda_{D}(1+\delta )}{j\omega \varepsilon_{f}}+\frac{R}{\sigma_{b}+j\omega \varepsilon_{b}}}$$

The value of α approaches a limiting value of $\alpha =\frac{1}{1+\delta }$ at ω→0, and another limiting value of $\alpha =\frac{\varepsilon_{b}\lambda_{D}}{\varepsilon_{f}R}$ at ω→0 for typical parameters in microfluidic systems.

The characteristic double-layer charge relaxation frequency of nonlinear electroosmotic streaming is defined as the particular one that makes the surface slip velocity on the polarizable interfaces drop by half. Due to the mathematical complexity, the analysis is made below the charge relaxation frequency of fluid bulk where conduction current dominates over displacement current within the liquid domain.

For $\sigma_{b}\to \infty$ and/or $\varepsilon_{b}\to \infty$ when ideally polarizable materials are used to construct such blocks, the efficiency of ICEO streaming at the diffusing interface is:

(21) $$\alpha_{\mathrm{conductor}}=\frac{Z_{\mathrm{EDL}}}{Z}=\frac{1}{1+\delta }\frac{1}{j\omega \tau_{\mathrm{RC}}}\;\text{with}\;\tau_{\mathrm{RC}}=\frac{\varepsilon_{f}W_{g}}{\sigma_{f}\lambda_{D}(1+\delta )}$$

Accordingly, the double-layer relaxation frequency is:

(22) $$f_{C}^{\mathrm{conductor}}=\frac{\sigma_{f}(1+\delta )}{2\pi \varepsilon_{f}}\frac{\lambda_{D}}{W_{g}}=O(f_{f-\mathrm{bulk}})\cdot \frac{\lambda_{D}}{W_{g}}<<O(f_{f-\mathrm{bulk}})$$

For $\sigma_{b}\to 0$, that is, the solid obstruction is made of pure dielectric material, as in the vicinity of the sharp corners of poly(dimethylsiloxane) (PDMS) microchannel junctions, *α* is dictated by the following expression if the dielectric layer capacitance dominates over the diffuse double layer ($\frac{\varepsilon_{b}}{R}\leq \frac{\varepsilon_{f}}{\lambda_{D}(1+\delta )}$):

(23) $$\alpha_{\mathrm{dielectric}}=\frac{\varepsilon_{b}\lambda_{D}}{\varepsilon_{f}R}\frac{1}{1+j\omega \frac{W_{g}\varepsilon_{b}}{\sigma_{f}R}}$$

The characteristic field frequency for double-layer charging on dielectric surfaces is:

(24) $$f_{C}^{\mathrm{dielectric}}=\frac{\sigma_{f}}{2\pi \varepsilon_{b}}\frac{R}{W_{g}}\approx \frac{\sigma_{f}}{2\pi \varepsilon_{b}}\;\sim O(f_{f-\mathrm{bulk}})$$

Which is on the order of the inverse charge relaxation time of liquid solution. The low frequency limiting value $\alpha_{\mathrm{dielectric}}^{\omega \to 0}=\frac{\varepsilon_{b}\lambda_{D}}{\varepsilon_{f}R}\leq \frac{1}{1+\delta }\leq 1$. Even at the sharp corners of nearly insulating dielectric blocks where R~O(λ_D_), the value of $\alpha_{\mathrm{dielectric}}^{\omega \to 0}$ is always less than that of $\alpha_{\mathrm{dielectric}}^{\omega \to 0}=\frac{1}{1+\delta }$ in DC limit, viz. $\alpha_{\mathrm{dielectric}}^{\omega \to 0}<\alpha_{\mathrm{conductor}}^{\omega \to 0}$. That is, due to the lower polarizability, the dielectric block always produces a lower flow velocity than metal electrodes. There are not so many restrictions on the applied field frequency with dielectric blocks, however, in that the occurrence of electrochemical ion relaxation is postponed to smaller time period of the applied AC voltage, which can even approach the charge relaxation time for onset of bulk ionic screening. This implies that, semiconductors of larger electrical impedance can produce stronger ICEO flow field within high frequency ranges than ideally polarizable surfaces due to a higher double-layer relaxation frequency $f_{C}^{\mathrm{dielectric}}<f_{C}^{\mathrm{conductor}}$. For this reason, we can make use of high-frequency ICEO induced next to leaky dielectric blocks to achieve sample mixing in microfluidics while a few of detrimental effects in low field frequencies are avoidable. It is noteworthy that the native surface charge chemically adsorbed on channel sidewalls is excluded in current work, in that the Coulomb force acting on the inherent Debye screening charge oscillates and time-averages to zero within each voltage period in AC fields.

### 2.5. Mixing Index

Mixing performance in dynamic flow condition is appraised in terms of sample concentration at the outlet, as characterized by the value of mixing index *γ*:

(25) $$\gamma =\left(1-\frac{\iint {}_{S}\left|c-0.5[\mathrm{mol}/m^{3}]\right|dA}{\iint {}_{S}0.5[\mathrm{mol}/m^{3}]dA}\right)\times 100\%$$

With this estimating approach, under the situation where perfect mixing emerges, *c* = 0.5 everywhere at the exit and γ=100%. On the contrary, if no mixing occurs, *c* = 1 or *c* = 0 at any position within the outlet plane and γ=0%. In this sense, the mixing index that is proposed here is phenomenally reasonable.

## 3. Results and Discussion

### 3.1. Vortex Streaming of Induce-Charge Electroosmosis Next to a Leaky Dielectric Block

An investigation on ICEO vortex streaming next to a leaky dielectric block lays the theoretical foundation for developing high-field-frequency electroosmosis micromixers. To begin with, we focus on the flow pattern of ICEO fluid motion around one triangular solid block under static condition (Figure 1a–c). The obstacle is embedded on the right side of a microfluidic channel that is full of saline solution, and has a sharp tip of radius of curvature *R* = 1 μm at the top area. The initial value for various parameters are chosen as: σ_f_ = 10^−3^ S/m, *C*_s_ = 0.8 F/m^2^, σ_b_ = 10^−7^ S/m, ε_b_ = 10ε_0_, L_c_ = 2 mm, W_c_ = 200 μm, L_b_ = 50 μm, W_b_ = 80 μm, A = 30 V.

#### 3.1.1. Effect of Solid Block Conductivity

On application of an AC voltage signal of suitable field parameters across the channel length direction, ions in the electrolyte follow the inclined electric field lines to move onto the surface of semiconductor block through electrophoretic transport (Figure 1a). After a typical double-layer relaxation time, a balance between electrostatic attraction and surface ion diffusion can be established. At the steady-state, a diffuse double layer of bipolar Debye screening charge is induced at the sharp material interface between the leaky dielectric block and electrolyte solution, and the electric field lines bend across the semiconductor surface right outside the Debye layer, forcing the induced double layer into a pair of ICEO vortex flow in opposite rotating directions near the leaky dielectric block (Figure 1b).

**Figure 2:**
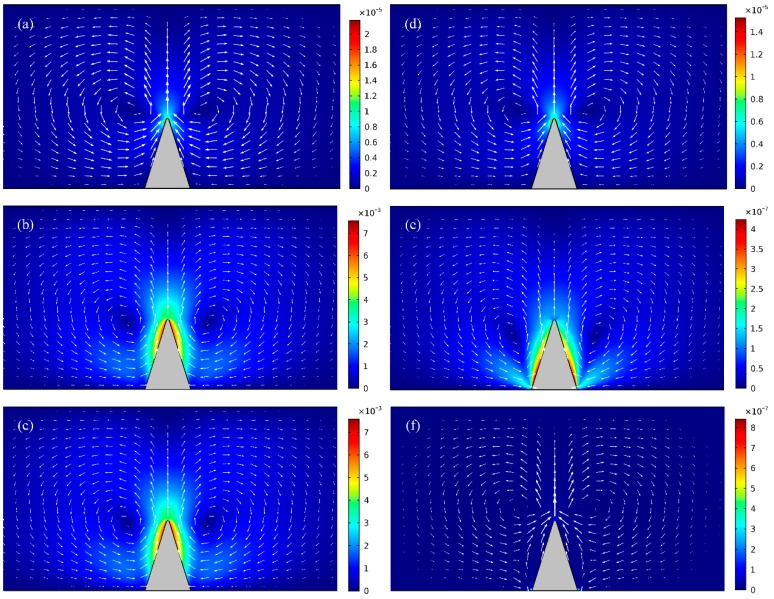
A surface and arrow plot of nonlinear electroosmotic flow field around an individual triangular block of different bulk conductivities at distinct field frequencies (unit: m/s). (**a**)–(**c**) under low-frequency limit (*f* = 10 Hz), (**a**) for σ_b_ = 0 S/m (insulating dielectric block of no internal charge carriers); (**b**) for σ_b_ = σ_f_ = 0.001 S/m (semiconductor block); (**c**) for σ_b_ = 10^4^σ_f_ = 10 S/m (ideally polarizable block). (**d**)–(**f**) at a much higher field frequency *f* = 100 kHz approaching the reciprocal charge relaxation time of fluid bulk, (**d**) for σ_b_ = 0 S/m; (**e**) for σ_b_ = σ_f_ = 0.001 S/m; (**f**) for σ_b_ = 10^4^σ_f_ = 10 S/m.

At first, numerical simulations are conducted for learning how the electrical conductivity of leaky dielectric block affects the ICEO flow field, while the AC voltage amplitude is fixed at A = 30 V. As shown in Figure 2a,d, for insulating dielectric block without bulk current flux, the vortex flow pattern keeps identical for *f* = 10 Hz and *f* = 100 Hz, and the magnitude of typical ICEO flow velocity decreases slightly from 22 μm/s to 15 μm/s, since the double-layer relaxation frequency $f_{C}^{\mathrm{dielectric}}=\frac{\sigma_{f}}{2\pi \varepsilon_{b}}=1.8\;\mathrm{MHz}$ for non-leaky material is raised by the large impedance of insulating solid block and even exceeds the Maxwell-Wagner polarization frequency of liquid solution $f_{f-\mathrm{bulk}}=\frac{\sigma_{f}}{2\pi \varepsilon_{f}}=225\;\mathrm{MHz}$.

Once the block is imperfectly insulated and has a finite electrical conductivity in equivalence to the ionic conductivity of the suspension medium, that is, σ_b_ = σ_f_ = 0.001 S/m, the flow field induced around the leaky dielectric obstruction increases to great extent (Figure 2b) in comparison to that actuated on insulating dielectric surfaces (Figure 2a) within low-frequency ranges. Nevertheless, at higher field frequencies, e.g., *f* = 100 KHz, magnitude of electroosmotic flow velocity decreases to even lower than 1 μm/s, which is too weak to be discernable in practical experiments (Figure 2e). 

As for a highly conductive solid obstruction of σ_b_ = 10^4^σ_f_ = 10 S/m, the block becomes an equipotential body, so the intensity of electroconvective vortex streaming attains an upper limit value at low field frequencies (Figure 2c). Under this situation, since there is almost no voltage that is dropped across the conducting block, the reciprocal double-layer relaxation time is given by the typical inverse RC time constant $f_{C}^{\mathrm{conductor}}=\frac{\sigma_{f}(1+\delta )}{2\pi \varepsilon_{f}}\frac{\lambda_{D}}{W_{g}}=173\;\mathrm{Hz}$ for induced double-layer charging on ideally polarizable surfaces, which is three orders of magnitude lower than the dispersion frequency of fluid bulk *f*_f-bulk_ = 225 kHz. Accordingly, the electroosmotic flow field decays rapidly with field frequency due to electrochemical ion relaxation, and the averaged fluid motion on the metal surface is further suppressed (Figure 2f) when compared to the case with semiconductor block (Figure 2e).

**Figure 3:**
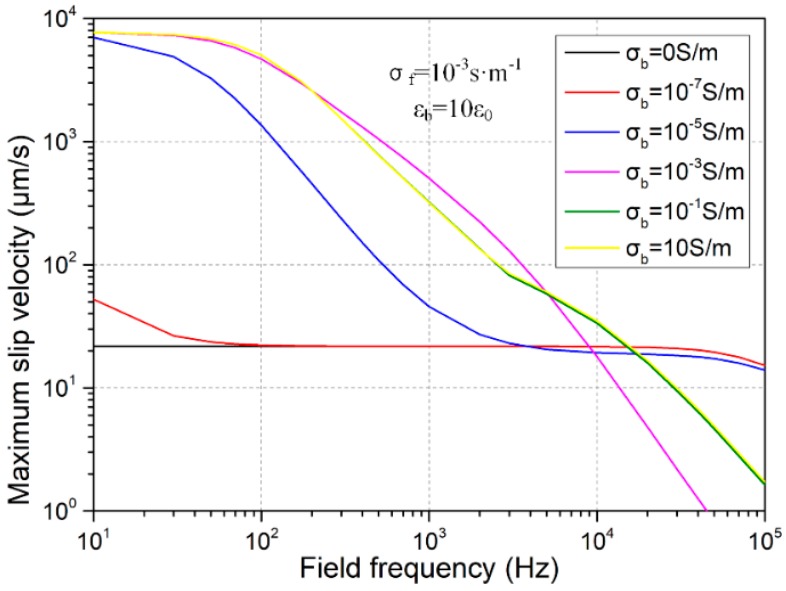
Frequency-dependent maximum ICEO slip velocity at the polarized phase interface for varying block conductivities.

The frequency-dependent electroosmotic flow velocity is quantified and exhibited in Figure 3. From the results with distinct bulk conductivities of the leaky dielectric block, it can be identified that the largest flow velocity is produced in the vicinity of conducting obstruction for conductors and semiconductors with conductivity larger or approaching that of the buffer solution (σ_b_ ≥ σ_f_ = 10^−3^ S/m), due to a high electrical polarizability of the solid material employed in the device design. As the applied field frequency increases, however, electroosmotic fluid motion decays quickly due to the small value of inverse RC time constant $f_{C}^{\mathrm{conductor}}=f_{f-\mathrm{bulk}}\frac{\lambda_{D}}{W_{g}}=173\;\mathrm{Hz}$, leading to trivial electroconvective streaming at *f* = 100 kHz (0.1–10 μm/s in Figure 3). On the other hand, for leaky dielectric block with electrical conductivity that is less than the suspension medium (σ_b_ < σ_f_ = 10^−3^ S/m), electroosmosis flow is greatly reduced in DC limit as compared to real semiconductors due to a lower polarizability of the solid obstacle, while no obvious dispersion process is manifested as the frequency increases, implying that higher flow velocity can be induced within high-frequency ranges with imperfect insulators of a small bulk conductivity. The utility of this deduction consists in that observable ICEO flow field can be actuated at high field frequencies (e.g., *f* = 100 kHz) with nearly insulating blocks, under the condition of which electrochemical reactions can be avoided to the best extent in the high frequency limit.

#### 3.1.2. Effect of Solution Conductivity

**Figure 4:**
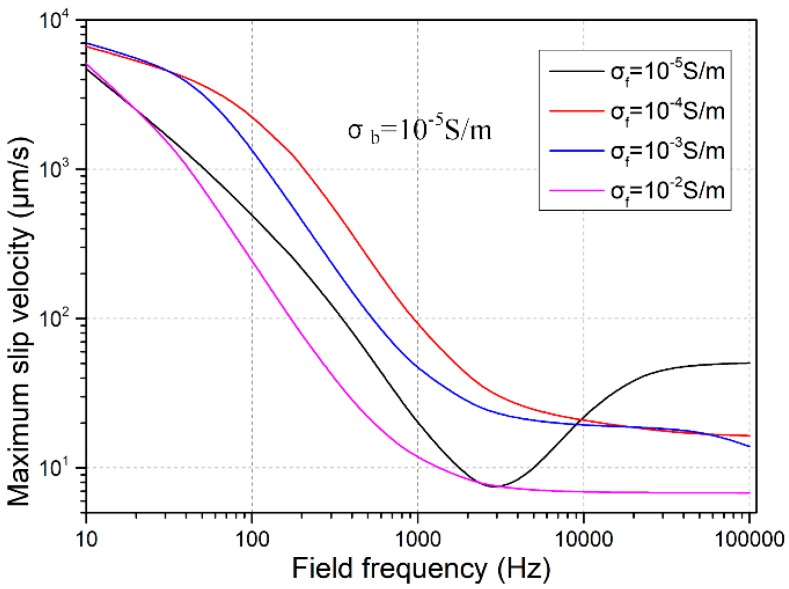
Influence of solution conductivity on ICEO vortex flow field as the block conductivity remains constant at 10^−5^ S/m.

The liquid conductivity may influence the electroconvective flow from three aspects. Firstly, as ion concentrations decreases, the Debye screening length increases, which will enhance ICEO flow field. Secondly, with higher ionic conductivity than the block conduction, the inverse double-layer relaxation time increases, this will reinforce ICEO flow velocity at higher field frequencies. Thirdly, a higher liquid conductivity decreases the relative polarizability of the semiconductor block, which makes the electroosmotic flow field slower in the low-frequency limit. In the presence of simultaneous action of these three mechanisms, as the field frequency increases, fluid motion due to the action of ICEO changes with distinct variation trends for different solution conductivities, as shown in Figure 4. According to the simulation results, the double-layer thickness plays the most dominating role over the issue on ionic charge relaxation time and/or block relative polarizability, in that the lowest liquid conductivity with the largest Debye length engenders the most efficient ICEO vortex flow at *f* = 100 kHz (the black line in Figure 4). In practice, however, since the liquid conductivity is usually of a given value and not easily adjustable, we should make attempt to reduce the bulk conductivity of leaky dielectric block to produce evident ICEO vortex streaming at high field frequencies for eliminating the possibility of Faradaic reactions and resulted bubble generations.

#### 3.1.3. Effect of Permittivity of Leaky Dielectric Obstruction

**Figure 5:**
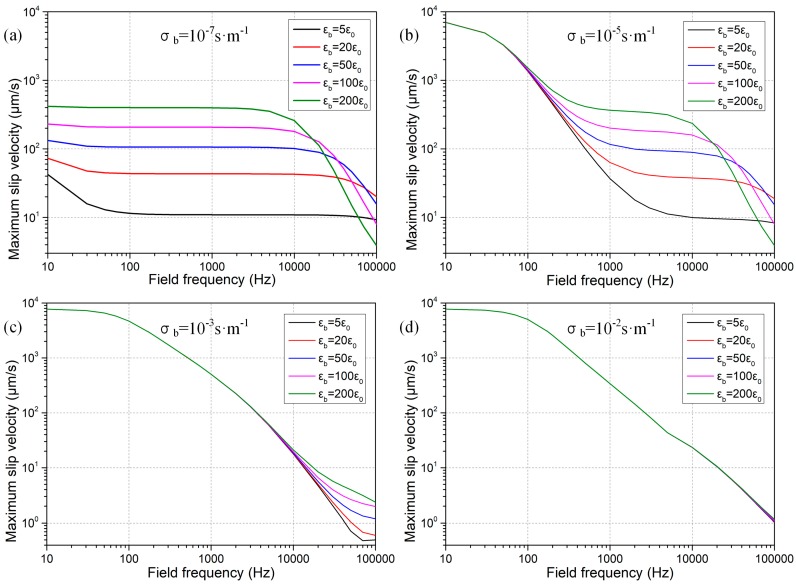
Effect of dielectric permittivity of leaky dielectric obstruction on the resulted ICEO flow field for a given value of liquid conductivity σ_f_ = 10^−3^ S/m for distinct block conductivities, (**a**) σ_b_ = 10^−7^ S/m; (**b**) σ_b_ = 10^−5^ S/m, (**c**) σ_b_ = 10^−3^ S/m, (**d**) σ_b_ = 10^−2^ S/m.

The specific value of dielectric permittivity of the semiconductor block exerts an influence on the ICEO flow field around the sharp geometrical tip as well. As for nearly insulating blocks with electrical conductivity lower than the ionic conductivity of the working fluids, the double-layer relaxation frequency $f_{C}^{\mathrm{dielectric}}=\frac{\sigma_{f}}{2\pi \varepsilon_{b}}$ decreases with an increasing permittivity of the solid obstacle, which makes the ICEO flow field at *f* = 100 kHz decay as the block polarizability becomes larger (Figure 5a,b). On the other hand, if the dielectric permittivity of solid block is sufficiently small, e.g., ε_b_ = 5ε_0_ (the black line in Figure 5a,b), ICEO flow velocity diminishes once again due to its excessively low polarizability. These results give rise to an optimal permittivity of ε_b_ = 20ε_0_ for nearly insulating semiconductor blocks, which can enable us to obtain efficient ICEO fluid motion in high-frequency ranges.

As the conductivity of solid obstruction is raised to higher values that equal the liquid ionic conductivity (Figure 5c), the small electrical impedance of the conductor protrusion renders the double-layer relaxation frequency much lower, so that ICEO fluid motion at *f* = 100 kHz is sharply decreased in comparison to that produced around nearly insulating obstruction, albeit a higher dielectric permittivity may enhance the flow field at high frequencies due to an increase in relative polarizability of the solid material with respect to that of the liquid suspension.

With further increase in concentration of charge carriers within the solid bulk that even surpasses the ionic conductivity of saline solution (Figure 5d), the high-frequency field-induced electroconvection becomes even weaker than that produced in the condition of equal conductivities (Figure 5c), and no permittivity effect on the fluid motion can be found any longer due to the domination of bulk conduction of the leaky dielectric obstacle (Figure 5d).

On the basis of above reasons, for the sake of avoiding those detrimental effects that are induced by Faradic charge-transfer reactions in low-frequency ranges, it is necessary to actuate ICEO vortex streaming at field frequency approaching the inverse charge relaxation time of the working fluid. On this premise, we should in priority choose to use nearly insulating materials (e.g., σ_b_ = 10^−7^ S/m) of a modest dielectric permittivity (ε_b_ = 20ε_0_) for constructing the semiconductor block that is embedded on channel sidewalls. 

### 3.2. Microfluidic Mixing with High-Frequency ICEO

We have made it clear what kind of leaky dielectric obstruction may give rise to appreciable ICEO fluid motion within the high-frequency range. With optimal electrical properties of the solid block, (σ_b_ = 10^−7^ S/m and ε_b_ = 20ε_0_), we than make a simulation analysis on the feasibility of this high-frequency vortex flow field of ICEO next to nearly insulating semiconductor blocks for sample mixing in microfluidics, in view of less severe electrochemical reactions occurring on the metal electrodes that are placed at the channel ends on both sides.

#### 3.2.1. Effect of the AC Voltage Signal on ICEO Fluid Motion

**Figure 6:**
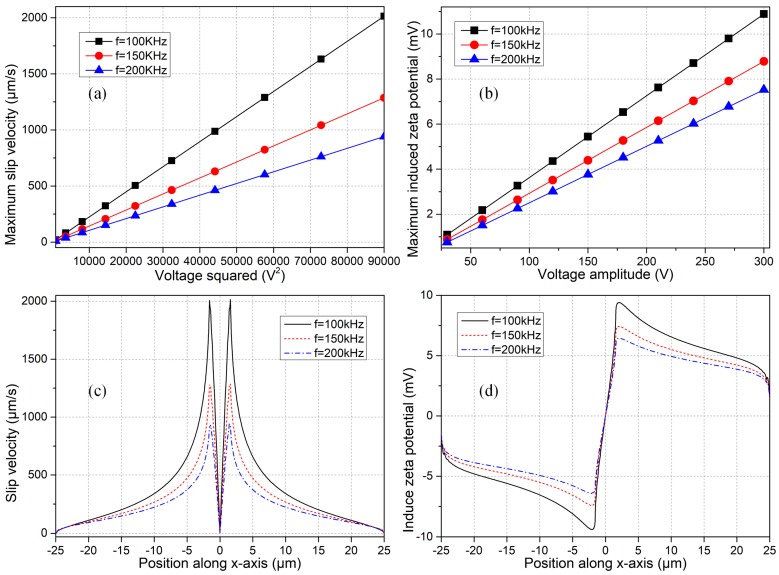
Effect of electrical field parameters on induced polarization and ICEO flow field next to a leaky dielectric block of σ_b_ = 10^−7^ S/m and ε_b_ = 20ε_0_. (**a**,**b**) Influence of voltage amplitude of the imposed AC signal on, (**a**) electroosmotic fluid motion; and (**b**) counterionic charge within the Debye layer, for different field frequencies in the context of high-frequency range (*f* ≥ 100 kHz). It is worthy to note that the induced zeta potential is even less than one thermal voltage of 25 mV on the surface of the nearly insulating semiconductor block. (**c**,**d**) Position-dependent distribution of, (**c**) ICEO slip velocity; and (**d**) double-layer voltage drop, on the block surface at a fixed voltage amplitude of A = 300 V for varying field frequencies (*f* ≥ 100 kHz).

Before coming to the subject of microfluid mixing, it is of great importance to study the influence of electric field parameters for driving sufficiently strong electroconvection even in the presence of a small zeta potential. The effect of voltage amplitude and field frequency of the applied AC signal on ICEO flow velocity and double-layer free charge is quantified and exhibited in Figure 6.

In the linear regime of diffuse charge dynamics, the fluid motion at different field frequencies has a quadratic growth trend with AC voltage amplitude, since one order of the applied harmonic potential difference induces the counterionic charge within the Debye layer (as evidenced by an increasing zeta potential with voltage in Figure 6b), and another order of electric field drives the induced charge cloud into nonlinear electroosmotic streaming. On another hand, for a given value of the AC voltage amplitude, ICEO fluid motion decreases with field frequency from 100 kHz to 200 kHz due to electrochemical ion relaxation that causes a lower zeta potential at higher frequencies (Figure 6b). However, the most salient feature consists in that, identical flow velocity can be produced at different field frequencies as long as the induced zeta potential is of a same value (even less than one thermal voltage of 25 mV), implying that a price of larger background field intensity has to be paid for higher field frequencies (Figure 6a,b). For instance, under the electric field parameters of *f* = 100 Hz and A = 120 V, *f* = 150 kHz and A = 150 V, as well as *f* = 200 kHz and A = 180 V, the electroosmotic flow velocity is almost of the same magnitude (Figure 6a) with an identical level of induced zeta potential around 4.2 mV (Figure 6b), so that our simulation model satisfies the condition of low-voltage limit. Besides, the Debye length of electrical double layer for liquid conductivity 0.001 S/m is 37.6 nm, while the size of block is 50 μm, the ratio of them is Λ = 37.6/50,000 = 0.000752 < 0.001. That is, the characteristic microscopic length scale of the microfluidic system, i.e., the double layer thickness, is more than three orders of magnitude smaller than its macroscopic counterpart, i.e., the obstruction span, so that the presumption of thin-layer approximation is apt for current analysis. For these reasons, this study stays safely within the Debye-Huckel limit, so the application of Equation (13) is appropriate and is in good accordance with that obtained by using the fully-coupled Possion-Nernst-Planck-Navier-Stokes model as well.

The position-dependent ICEO slip velocity is shown in Figure 6c for a fixed voltage amplitude of A = 300 V. The fluid motion vanishes right on the sharp tip of the leaky dielectric block for varying field frequencies, due to a geometric symmetry in the distribution of induced zeta potential with respect to the center of solid surface (Figure 6d). At the same time, the slip flow attains a peak value at the edge of the block on both sides with a small distance from the sharp tip (Figure 6c), due to the action of inhomogeneous double-layer charging modes at the solid/electrolyte interface (Figure 6d).

As a result, by making a flexible adjustment in the voltage amplitude of applied AC signal, it is possible to actuate evident ICEO vortex flow in the transverse direction, even in the presence of a quite small induced zeta potential (less than 10 mV) at distinct field frequencies (*f* ≥ 100 kHz) in the context of a high-frequency range.

#### 3.2.2. ICEO Micromixer with Sharp Protrusions of Leaky Dielectric Blocks

##### Mixer with One Pair of Solid Obstacles

**Figure 7:**
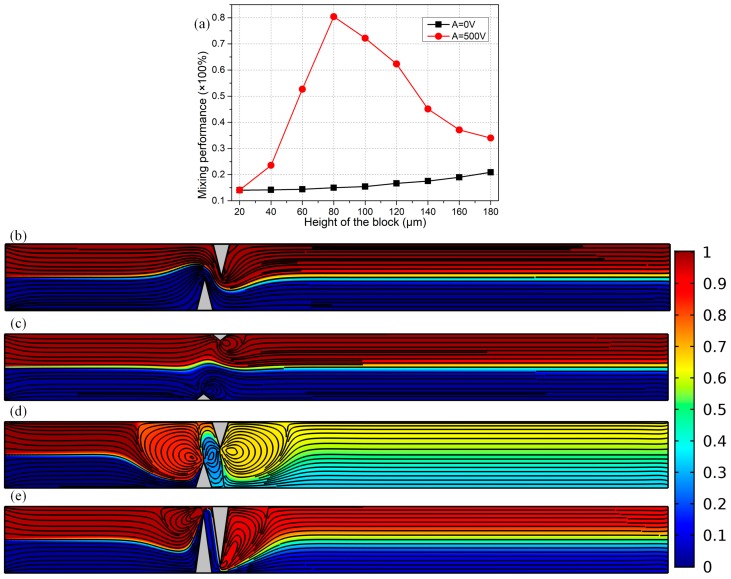
In the context of a simple micromixer structure using merely one pair of leaky dielectric blocks positioned on opposing channel sidewalls, simulation results of mixing process with different width of the semiconductor blocks. (**a**) The effect of block width on the mixing performance with (A = 500 V) and without ICEO (A = 0 V); (**b**)–(**e**) A surface plot of fluorescence concentration (unit: mol/m^3^) and a streamline plot of flow field in the micromixer taking advantage of transversal ICEO perturbation flow in perpendicular orientation to the axial two co-flowing laminar streams for different block height, (**b**) at W_b_ = 100 μm without ICEO (A = 0 V); (**c**) at W_b_ = 20 μm with A = 500 V; (**d**) at W_b_ = 80 μm with A = 500 V; (**e**) at W_b_ = 180 μm with A = 500 V.

Based upon the preceding analysis on the fundamental flow behavior of ICEO next to one leaky dielectric block under different conditions, it is interesting to make use of such kind of high-frequency electroosmotic flow that is produced around nearly insulating obstructions for achieving sample mixing in microchannel embedded with semiconductor protrusions on both sidewalls (Figure 1d). To begin with, we first focus on the effectiveness of a simple device structure with merely one pair of leaky dielectric blocks positioned on opposing channel sidewalls in stirring fluidic samples, as shown in Figure 7.

##### A. Optimization of Block Width

For achieving sample mixing, we deliberately raise the applied voltage amplitude to *A* = 500 V in the numerical calculation, in which the induced zeta potential is still less than the value of double thermal voltage 50 mV at *f* = 100 kHz. For such, it is reasonable to invoke the linear asymptotical approximation. The leaky dielectric block has an electrical conductivity of σ_b_ = 10^−7^ S/m and dielectric permittivity of ε_b_ = 20ε_0_, the inlet flow rate is set to u_0_ = 100 μm/s in the early stage. The fluorescence analytes of 40 μm in diameter have a thermal diffusivity of *D* = 10^−11^ m^2^/s from Einstein relation.

The length of triangular obstacle is fixed L_b_ = 50 μm, which is one-fourth of the channel width W_c_ = 200 μm. Under this situation, it is very useful to conduct an optimization of the device geometry, e.g., to study the effect of block width on the resulted mixing performance, with the detailed simulation results presented in Figure 7. According to Figure 7a, in the absence of ICEO vortex flow with the function generator turned off (A = 0 V), the mixing is merely due to the action of diffusive mass transfer in a concentration gradient across the two-phase contact interface (Figure 7b), resulting in a poor mixing performance of less than 20% at the channel exit whatever the width of semiconductor is.

On the contrary, switching on the signal generator outputting a harmonic voltage wave of amplitude A = 500 V and field frequency *f* = 100 kHz, turbulent ICEO vortex flows in reverse rotating directions are induced by the two polarizable semiconductor blocks, which intersect perpendicularly with the co-flowing laminar streams in the horizontal direction coming from the two upstream channel inlets, resulting in helical flow streamlines marching along the channel length direction toward the downstream outlet (Figure 7c–e). In view of this, the field-induced chaotic electroconvection accelerates the rate of mass exchange across the diffusing phase interface by boosting convective mass transfer in the lateral direction.

However, the degree of mixing enhancement can make a difference as the width of the leaky dielectric blocks is changed. With a quite small obstruction width W_b_ = 20 μm = W_c_/10 (Figure 7c), since the actuating range of ICEO vortex flow is merely confined to the vicinity of thin blocks, the transversal perturbation streamlines cannot act well on the two-phase flow interface, giving rise to a poor mixing performance of 14%, which is comparable to that in the absence of electroconvective streaming (Figure 7a,b). As the obstacle width approaches the channel width, e.g., W_b_ = 180 μm (Figure 7e), ICEO whirlpools are suppressed to a great extent by the viscous boundary layer effects on opposite channel sidewalls. Within the intermediate range of the size of the solid block, e.g., W_b_ = 80 μm (Figure 7d), not only the ICEO fluid motion can be fully developed with a broad actuating range, but there is enough spacing for neighboring vortex flows to actively interact and exert appreciable hydrodynamic stress on the diffusing interface along the channel transverse direction as well. In this way, the resulted helical flow streamlines give rise to a decent mixing performance of γ = 80% (Figure 7d) at the channel exit.

For these reasons, the mixing process is highly dependent on the ratio of block size to channel width, that is, the value of *α* = W_b_/W_c_, which is confined within the range 0 < *α* < 1 by geometrical constraints. For blocks of a quite small size (*α* → 0), the negligibly small actuating range of ICEO vortex fails to make contact with the diffuse interface, resulting in unsatisfactory mixing efficiency (Figure 7a). As the solid blocks tend to fully obstruct the incoming laminar streams (*α* → 1), the viscous boundary layers at the solid/electrolyte interface restrain the healthy development of these transversal vortex flows from ICEO, which again lowers the mixing performance to great extent (Figure 7a). Accordingly, there always exists an optimal block width for sample mixing in the microchannel, in terms of a peak point of mixing performance at W_b_ = 80 μm (*α* = 0.4), according to the calculation results in Figure 7a.

##### B. Inter-Block Separation

**Figure 8:**
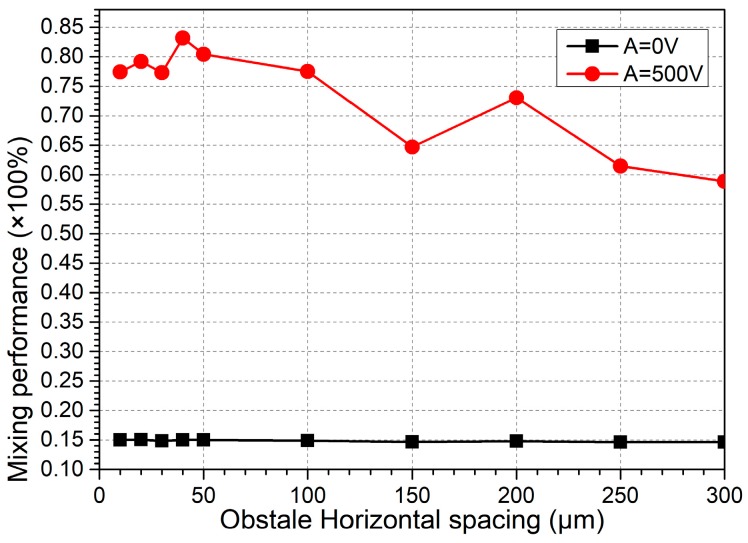
A numerical study of the influence of the horizontal distance between adjacent opposing blocks on the mixing performance.

With the best block width W_b_ = 80 μm for the basic device structure (Figure 7d), we then need to know how the gap distance L_bb_ between the adjacent obstructions that are placed on opposite channel sidewalls affects fluid mixing in the micromixer. As shown in Figure 8, as the inter-block separation L_bb_ increases from 0 μm to 300 μm, the mixing performance decreases roughly on the whole. The reason behind this phenomenon is that the neighboring ICEO vortex flows in reverse rotating directions can actively interact with one another at a sufficiently small horizontal gap L_bb_ between adjacent opposing obstructions, which is beneficial to produce excellent fluid mixing. As the inter-block separation increases, however, neighboring micro-vortices tend to be mutually independent, which indirectly stretches the zigzag particle course along channel length direction and consequently lowers the mixing efficiency to some extent (Figure 8).

So, in practical applications, we ought to select an optimal inter-block separation L_bb_ to induce the ideal mixing behavior of incoming nanoparticle samples in ICEO micromixers, and the difficulty in microfabrication process must be taken into consideration for establishing a sufficiently small gap size at the same time. In this sense, L_bb_ = 40 μm is chosen as the most suitable inter-block gap size for subsequent simulation analysis.

##### C. Effect of Voltage Amplitude and Inlet Flow Rate on Sample Mixing

Previous analysis has concentrated on the effect of geometry configuration on the mixing behavior in the presence of given values of voltage amplitude A = 500 V and inlet flow rate *u*_0_ = 100 μm/s. In this section, however, armed with the optimized geometric size of the device structure (L_bb_ = 40 μm and W_b_ = 80 μm) revealed in preceding analysis, we make an attempt to interpret how these two parameters influence the mixing behavior for engendering better performance.

**Figure 9:**
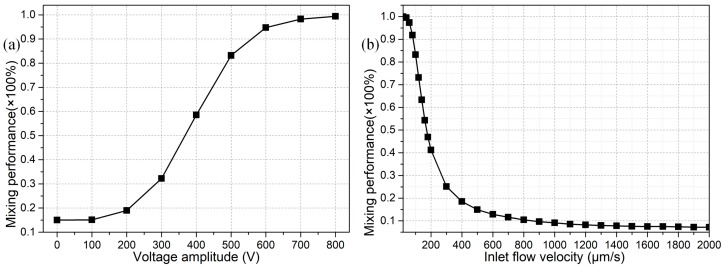
For given values of field frequency *f* = 100 kHz, inter-block separation L_bb_ = 40 μm and obstruction width W_b_ = 80 μm, (**a**) the effect of voltage amplitude on the mixing performance at an inlet flow rate of *u*_0_ = 100 μm/s; and (**b**) influence of inlet flow velocity on the fluid mixing behavior at A = 500 V.

As shown in Figure 9a, even in the high field frequency range *f* = 100 kHz, voltage amplitude of the applied AC signal plays an important role in the stirring process, due to a second-order voltage dependence of ICEO on polarizable solid surfaces that can greatly enhance the vortex flow in the transverse direction at higher electric field strength for the improvement of mixing performance (Figure 9a). In reality, however, since these is an upper limit in the magnitude of harmonic voltage signal as set by the practical issue of Joule medium heating and any other non-equilibrium surface-electro phenomenon (NESP), we preferentially choose an intermediate value of voltage magnitude A = 500 V that gives rise to a moderate mixing efficiency of 83% at an inlet flow velocity *u*_0_ = 100 μm/s (Figure 9a).

The helical flow streaming in the forward direction facilitates the rectification of fluid mixing behavior, and is originated by an orthogonal crossover of the axial pressure-driven flow with the transverse electroconvection around the leaky dielectric blocks. Accordingly, the flow velocity of incoming laminar streams with a parabolic profile can also exert an impact on the mixing efficiency, as indicated in Figure 9b. On one hand, for a low inlet flow velocity of a small hydrodynamic Reynolds number, there is plenty of time for incoming fluids to blend along the channel length direction as they are slowly transported toward the downstream exit, resulting in a fairly good mixing efficiency at the price of lowering the throughput. On another hand, with a higher inlet flow rate of a larger Reynolds number, there are less chances for the helical perturbation flows to rotate among these semiconductor blocks, and consequently the mixing efficiency is suppressed, while we can get a high-throughput at the channel exit, as validated by the calculation results in Figure 9b, where the fluid mixing behavior is worsened with an increase in the inlet flow velocity. Accordingly, to realize high-throughput sample mixing with ICEO next to semiconductor blocks, we should embark on tackling the matter of how to mingle fluid samples at a relatively large Reynolds number.

##### Design of Integrated Microfluidic Mixers

**Figure 10:**
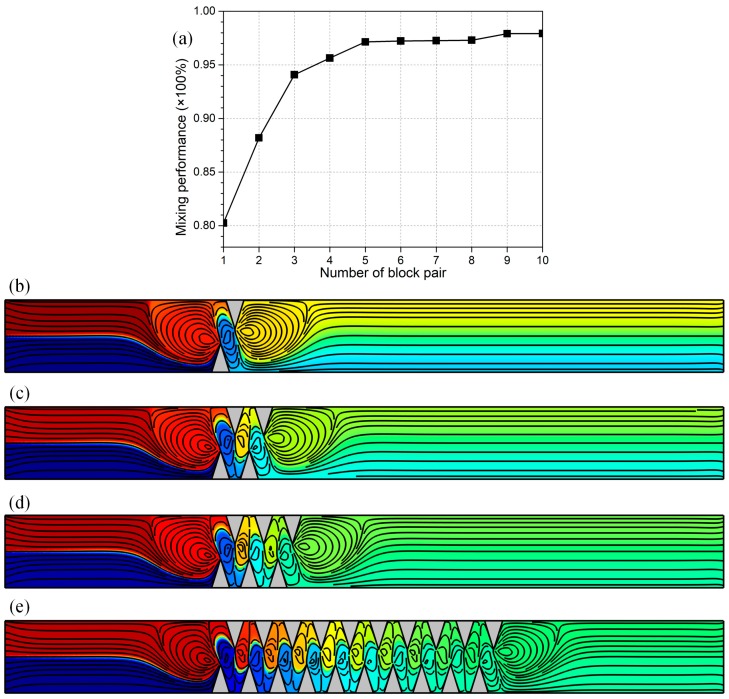
Development of integrated microdevices for high-flux sample mixing in microfluidics, in which a series of semiconductor blocks are deposited on opposite channel sidewalls in a staggered way, for given values of *u*_0_ = 200 μm/s, A = 700 V, *f* = 100 kHz, W_b_ = 80 μm and L_bb_ = 40 μm. (**a**) Influence of number of the block pair on mixing performance of incoming fluorescein nanoparticles. (**b**–**e**) A surface plot of sample concentration distribution (mol/m^3^) and streamline plot of flow field in the microchannel with different number of obstruction pair, (**b**) one pair; (**c**) two pairs; (**d**) three pairs; and, (**d**) ten pairs.

To address the issue of sample mixing at higher throughputs, a more advanced structure of ICEO micromixer is developed, in which a series of leaky dielectric blocks are sequentially configured on opposing channel sidewalls in a staggered way, as shown in Figure 10b–e. Other geometric size keeps identical with the optimal values found in preceding analysis, i.e., *f* = 100 kHz, L_b_ = 50 μm, W_b_ = 80 μm and L_bb_ = 40 μm, whereas the inlet flow rate is increased from *u*_0_ = 100 μm/s to 200 μm/s to test the feasibility of this integrated device design in generating high-throughput sample mixing. In the meantime, the applied voltage amplitude is slightly increased from A = 500 V to 700 V to accommodate the rise in Reynolds number.

To simply the relevant analysis, we primarily consider the effect of number of pairs of semiconductor blocks deposited in sequence on opposite channel sidewalls. As exhibited in Figure 10a, with increasing number of opposing block pairs, the mixing behavior is improved sharply at the early time (Figure 10b–d). However, as the number of obstruction pair reaches *n* = 5, the mixing performance attains a plateau of *γ* = 97% in the range of large values of *n* (5 ≤ *n* ≤ 10), and mixing behavior will not improve with further increase of *n* any longer (Figure 1e). This implies that it is not a cost-efficient approach to enhance electro-convective mixing by simply increasing the number of block pairs embedded on opposing channel sidewalls. On the contrary, we should seek for the most appropriate amount of semiconductor protrusions for this integrated microfluidic mixer by conducting computational optimization in advance, which not only saves the expense for the fabrication process, but is able to generate the peak mixing performance as well, e.g., *n* = 5 under current situation (Figure 10a).

It is noteworthy that, in order to produce ideal mixing performance at a relatively high inlet flow rate *u*_0_ = 200 μm/s, we have employed a large background electric field strength *E*_0_ = 3500 V/cm in the integrated micromixer, which can give rise to appreciable temperature elevation through Joule heating, and thereby bring about some intractable problems and detrimental effects to both the working fluid and device structure. Accordingly, as one of the most important precautions before experimentation, the liquid conductivity should be made small enough for avoiding the induction of a high temperature rise in the integrated channel design.

##### Mixing with Smaller Voltages

**Figure 11:**
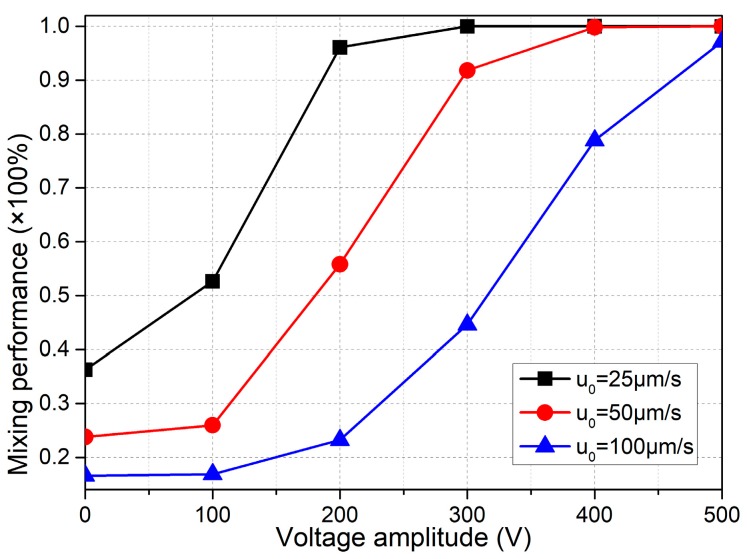
Voltage-dependent mixing performance for different inlet flow velocity, in which the applied electric field strength is no more than *E*_0_ = 2500 V/cm.

For practical application, we then have a check on the possibility of the integrated design with *n* = 4 in causing effective mixing for lower applied voltages in the range of A = 0–500 V. As presented in Figure 11, in order for the microfluidic mixer to work efficiently at a field strength below *E* ≤ 2500 V/cm, the inlet flow velocity must be no more than *u*_0_ = 100 μm/s, or else voltages larger than 500 V have be employed to realize ideal mixing behavior as *u*_0_ is beyond 100 μm/s.

If the critical field intensity *E*_C_ is defined as the one that results in a mixing performance of no less than 90%. Then, from Figure 11, E_C_ for *u*_0_ = 25 μm/s, *u*_0_ = 50 μm/s, and *u*_0_ = 100 μm/s is 200 V, 300 V and 500 V, respectively. So, a larger value of *E*_C_ is required to accommodate the enhancement in inlet flow rate. In fact, as the Reynolds number increases, there is less time for ICEO vortex flow actuated in the lateral direction to act on the incoming sample flow which quickly passes through the microchannel along the length direction, and consequently for larger inlet flow rate, we have to enhance ICEO flow velocity by raising the applied voltage for obtaining not bad mixing performance.

As a result, when the inlet flow rate increases, the critical voltage for onset of ideal mixing behavior is enhanced greatly, and therefore the issue of Joule medium heating in conducting electrolyte full of charge carriers is inevitable. For addressing its negative impact on the device performance, a good choice of precaution before experimentation is to dilute the liquid solution for lowering the ionic strength as far as possible if we need to conduct sample mixing at a relatively large inlet flow rate.

## 4. Conclusions

In short summary, we have presented results from both impedance analysis and direct numerical simulation to account for the phenomenon of ICEO slip flow due to the Coulomb force acting on the field-induced bipolar Debye screening charge on polarizable surfaces of leaky dielectric blocks of arbitrary electrical properties, as well as the resulted mixing behavior of incoming fluorescence nanoparticles under a delicate combination of a series of ICEO vortex flows in reverse rotating directions along the channel length direction. Our mathematical analysis indicates that induced-charge electrokinetics can be actuated at high field frequencies even approaching the reciprocal charge relaxation time of the bulk solution. This kind of high-frequency electroosmotic vortex is directly related to several important on-chip applications, including sample mixing and characterization in microfluidic devices. Rotating electrokinetic micro-vortices may be either profitable (mixing) or detrimental (pumping), and the physical insights on high-frequency ICEO adjacent to semiconductor obstructions recounted in this work can guide the design of flexible electrokinetic frameworks to either strengthen or restrain them. Plausible extensions of current works include the investigation in the presence of some large-voltage effects, including bipolar Faradaic reactions, nonlinear surface capacitance, inhomogeneous surface conduction, and related bulk concentration polarization, as well as chaotic electroconvection due to a positive current feedback near the extended space charge layer in ion-depletion zone. Under such conditions, linear asymptotic analysis employed in current work loses effect and the renewed mathematical formulations have to take into account the action of nonlinear diffuse charge dynamics at different Dukhin numbers. It is firmly believed that the high-frequency ICEO will bring about exciting inspirations in the interdisciplinary research fields of AC electrokinetics, analytical chemistry and micro/nanofluidics, and provide precious opportunities for causing active interactions between biochemical engineers, fluid mechanics scientists, and applied mathematicians in the near future.
